# Platelet-Rich Plasma in Aesthetic Dermatology: Current Evidence and Future Directions

**DOI:** 10.7759/cureus.66734

**Published:** 2024-08-12

**Authors:** Joshua Asubiaro, Felix Avajah

**Affiliations:** 1 Pharmaceutical Medicine, Accellacare, ICON plc, London, GBR; 2 Pharmaceutical Medicine, Bioluminux, Milton Keynes, GBR; 3 Aesthetic Medicine, JS Medical Aesthetics, Billericay, GBR; 4 Psychiatry, Rhodes Wood Hospital, Elysium Healthcare, Brookmans Park, GBR; 5 Aesthetic Medicine, The Medical Aesthetics Clinic and Wellness Centre Ltd, Lagos, NGA; 6 Aesthetic Medicine, Dr Therapy Beauty Clinic And Spa, Lagos, NGA

**Keywords:** fat grafting, wound healing, hair restoration, skin rejuvenation, aesthetic dermatology, platelet-rich plasma

## Abstract

Platelet-rich plasma (PRP) has emerged as a promising treatment in aesthetic dermatology. This systematic review aims to evaluate the current evidence for PRP applications in skin rejuvenation, hair restoration, wound healing, and fat grafting. Following Preferred Reporting Items for Systematic Reviews and Meta-Analyses (PRISMA) guidelines, a comprehensive literature search was conducted across multiple databases and 13 studies meeting the inclusion criteria were selected for review. The Mixed Methods Appraisal Tool (MMAT) was used to assess the quality of included studies. The reviewed studies consistently reported positive outcomes for PRP across various applications. In skin rejuvenation, significant improvements in collagen density and overall skin appearance were observed. For hair restoration, studies showed mean increases of 18-27.7 hairs/cm² in treated areas. PRP demonstrated efficacy in accelerating wound healing across various wound types, including chronic ulcers. In fat grafting, PRP was associated with improved graft survival and integration. Patient satisfaction was generally high across all applications. However, there was significant heterogeneity in PRP preparation methods and treatment protocols among studies. This systematic review provides evidence supporting the efficacy of PRP in aesthetic dermatology, particularly in skin rejuvenation, hair restoration, wound healing, and fat grafting. PRP demonstrates a favorable safety profile across applications. However, the variability in study designs and PRP protocols highlights the need for standardization. Future research should focus on large-scale randomized controlled trials with standardized protocols and longer follow-up periods to solidify the evidence base for PRP in aesthetic dermatology.

## Introduction and background

Platelet-rich plasma (PRP) has emerged as a novel and promising therapeutic modality in the field of aesthetic dermatology [1]. This autologous blood product, rich in growth factors and cytokines, has garnered significant attention for its potential to promote tissue regeneration, enhance collagen synthesis, and stimulatecellular proliferation [2,3]. The application of PRP in aesthetic dermatology has expanded rapidly over the past decade, with practitioners exploring its efficacy in addressing a wide range of dermatological concerns, from facial rejuvenation to hair restoration [4,5].

The principle underlying PRP therapy lies in harnessing the body's natural healing mechanisms. By concentrating platelets from the patient's own blood and reintroducing them to targeted areas, PRP aims to stimulate tissue repair and regeneration [6]. This process is mediated by the release of various growth factors, including platelet-derived growth factor (PDGF), transforming growth factor-β (TGF-β), vascular endothelial growth factor (VEGF), and epidermal growth factor (EGF), among others [7]. These growth factors play crucial roles in cell proliferation, differentiation, and angiogenesis, which are essential processes in tissue regeneration and rejuvenation [8].

In the realm of aesthetic dermatology, PRP has been investigated for its potential benefits in several key areas. Facial rejuvenation studies have explored the use of PRP for improving skin texture, reducing fine lines and wrinkles, and enhancing overall facial appearance [9,10]. The application of PRP in treating atrophic acne scars has shown promise, both when used alone and in combination with other treatments such as microneedling or fractional laser therapy [11,12]. Hair restoration, particularly for androgenetic alopecia and other forms of hair loss, has gained traction with several studies reporting positive outcomes in terms of hair density and thickness [13,14].

Moreover, researchers have investigated PRP's potential to improve overall skin quality, elasticity, and hydration [15]. The synergistic effects of combining PRP with other aesthetic treatments such as microneedling, laser therapy, and dermal fillers have been a subject of exploration, with some studies suggesting enhanced outcomes compared to monotherapies [16,17]. Despite the growing popularity of PRP in aesthetic dermatology, the scientific evidence supporting its efficacy and safety remains heterogeneous and, in some cases, controversial [18]. Variations in preparation methods, including centrifugation protocols and activation techniques, have led to inconsistent PRP compositions across studies [19]. Furthermore, the lack of standardization in treatment protocols, including injection techniques, treatment intervals, and the number of sessions, has contributed to variable results [20].

The long-term effects and optimal treatment regimens for different aesthetic indications are yet to be fully elucidated [21]. While some studies report sustained improvements in skin quality and hair growth, others suggest the need for maintenance treatments to preserve the benefits [22,23]. Additionally, the mechanisms by which PRP exerts its effects in different aesthetic applications are not fully understood, necessitating further investigation into the molecular and cellular processes involved [24].

Safety considerations surrounding PRP therapy in aesthetic dermatology have also been a subject of discussion. While generally considered safe due to its autologous nature, there have been reports of adverse events such as erythema, edema, and bruising at injection sites [25]. The potential for more serious complications, although rare, underscores the importance of proper patient selection, aseptic technique, and adherence to standardized protocols [26].

As the field of aesthetic dermatology continues to evolve, there is a pressing need for a comprehensive evaluation of the current evidence surrounding PRP therapy. This systematic review aims to address this gap by critically appraising the available literature on PRP in aesthetic dermatology, focusing on its efficacy, safety (short-term side effects and potential long-term complications), and potential future directions. By synthesizing the existing evidence, identifying knowledge gaps, and highlighting emerging trends, this review seeks to provide valuable insights for clinicians, researchers, and policymakers in the field of aesthetic dermatology. The findings of this systematic review will not only inform clinical practice but also guide future research efforts. While PRP therapy represents a promising frontier in aesthetic dermatology, offering the potential for natural and autologous tissue rejuvenation, the need for rigorous scientific evaluation and standardization of protocols remains paramount. By elucidating the strengths and limitations of current evidence and assessing the effectiveness of PRP therapy in different aesthetic dermatology applications, including (a) Facial rejuvenation, (b) Scar treatment, (c) Hair restoration, and (d) Overall skin quality improvement, this review aims to contribute to the development of evidence-based guidelines for the use of PRP in aesthetic dermatology. Furthermore, it will highlight areas requiring further investigation, potentially stimulating new research directions and fostering innovation in this rapidly evolving field.

## Review

Methods

This systematic review was conducted in accordance with the Preferred Reporting Items for Systematic Reviews and Meta-Analyses (PRISMA) guidelines [27]. The details of the PICO (population, intervention, control, and outcomes) framework are given in Table 1.

**Table 1:** PICO Framework PICO: population, intervention, control, and outcomes

PICO	Concept	Detail
Population	Adult patients (typically 18 years and older) seeking aesthetic dermatological treatments	Individuals with signs of facial aging (e.g., fine lines, wrinkles, loss of skin elasticity) Patients with acne scars or other types of atrophic scars Individuals experiencing hair loss, particularly androgenetic alopecia Patients seeking overall skin rejuvenation or improvement in skin quality Both male and female patients Various skin types and ethnicities
Intervention	Platelet-rich plasma (PRP) therapy	PRP injections for facial rejuvenation PRP applications for scar treatment PRP therapy for hair restoration PRP treatments for overall skin quality improvement
Comparison	Standard treatments, placebo, or no treatment	Standard care or conventional treatments for each indication (e.g., topical treatments, chemical peels, laser therapy) No treatment (wait-list control) Different PRP preparation methods or application techniques
Outcome	Efficacy and safety	Efficacy outcomes: Improvement in skin texture and quality (measured by standardized scales or imaging techniques) Reduction in the appearance of fine lines and wrinkles Improvement in skin elasticity and firmness Reduction in the visibility of scars Increase in hair density, thickness, and growth rate Patient satisfaction and quality of life measures Duration of effects and need for maintenance treatments Safety outcomes: Incidence and severity of adverse events (e.g., erythema, edema, bruising) Serious adverse events or complications Long-term safety profile Patient-reported discomfort or pain during and after treatment

Outcome Measures

Primary outcome measures included the clinical efficacy of PRP in various aesthetic dermatology applications with safety and adverse events. Secondary outcome measures included patient satisfaction, histological changes, long-term efficacy, and comparison with other treatments.

Search Strategy and Study Selection Process

A comprehensive literature search was conducted using electronic databases including PubMed, Embase, Cochrane Library, and Web of Science. The search strategy incorporated relevant keywords and Medical Subject Headings (MeSH) terms related to platelet-rich plasma and aesthetic dermatology. The search terms included: "platelet-rich plasma," "PRP," "skin rejuvenation," "hair restoration," "alopecia," "wound healing," "fat grafting," "aesthetic dermatology," and "cosmetic dermatology." The search was conducted in 2024. PICO Framework along with the corresponding MeSH terms are given in Table 2.

**Table 2:** PICO Components with Corresponding MeSH Terms PICO: population, intervention, control, and outcomes; MeSH: Medical Subject Headings

PICO Component	Description	MeSH Terms
Population	Adult patients undergoing aesthetic dermatology procedures	"Adult" [Mesh],"Dermatology" [Mesh],"Cosmetic Techniques" [Mesh].
Intervention	Platelet-rich plasma (PRP) treatment	"Platelet-Rich Plasma" [Mesh],"Regenerative Medicine" [Mesh].
Comparison	Standard treatments, placebo, or no treatment	"Standard of Care" [Mesh],"Therapeutic Equivalency" [Mesh].
Outcome	Efficacy in skin rejuvenation, hair restoration, wound healing, and fat grafting; patient satisfaction; safety	"Treatment Outcome" [Mesh],"Rejuvenation" [Mesh],"Hair Follicle" [Mesh],"Wound Healing" [Mesh],"Adipose Tissue/transplantation" [Mesh],"Patient Satisfaction" [Mesh],"Safety" [Mesh].

Inclusion criteria encompassed randomized controlled trials (RCTs), prospective and retrospective cohort studies, case-control studies, and systematic reviews investigating the use of PRP in aesthetic dermatology applications, published in English between January 2010 and April 2024. We included primarily human studies on the use of PRP in aesthetic dermatology. In studies that had both human and animal components, we included them if the human component provided valuable information relevant to our review objectives. This decision was made through mutual discussion among all reviewers. Case reports and case series with fewer than 10 participants, conference abstracts, and non-peer-reviewed literature were excluded.

**Figure 1:**
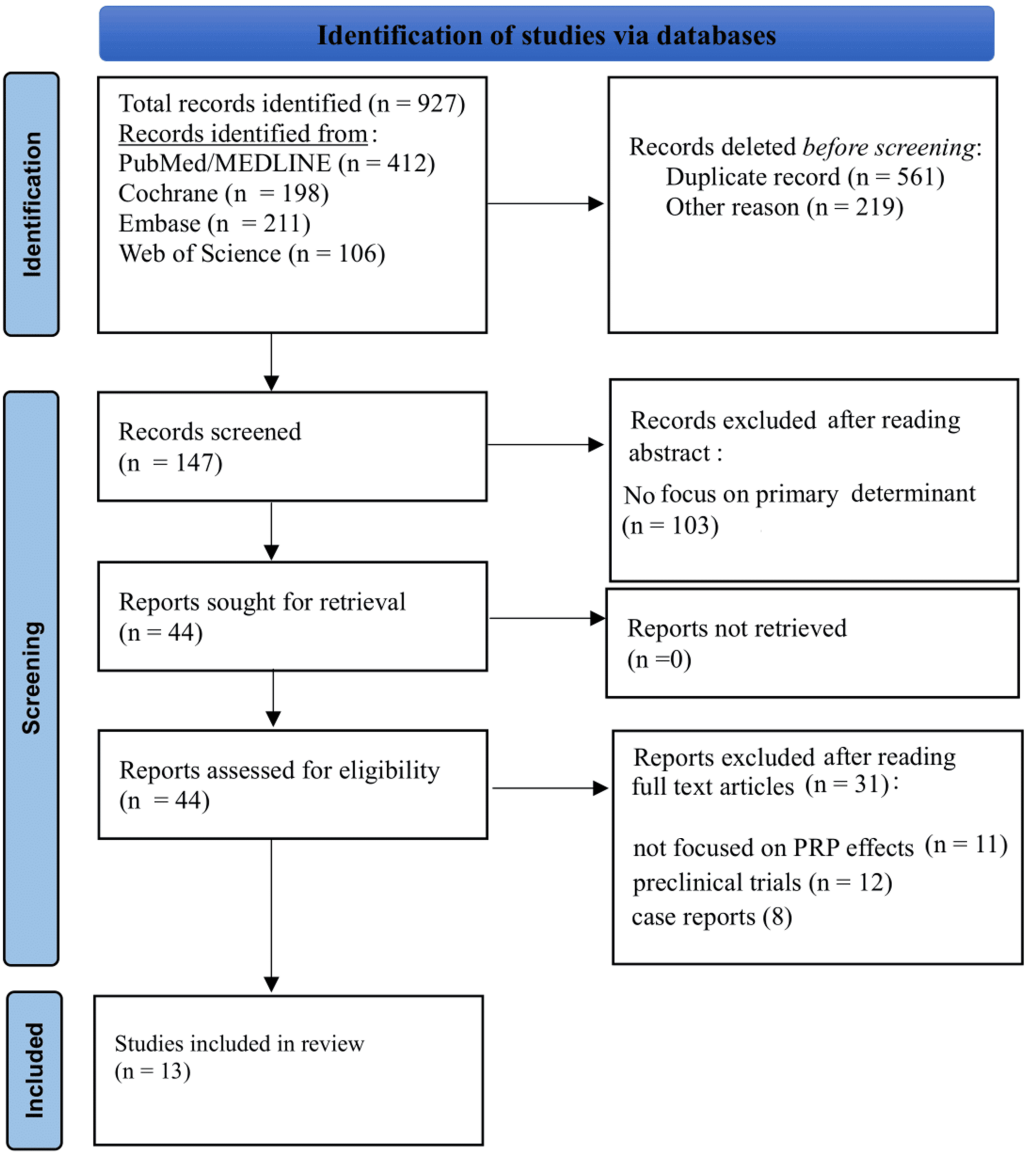
PRISMA Flowchart PRISMA: Preferred Reporting Items for Systematic Reviews and Meta-Analyses

The authors screened titles and abstracts, followed by a full-text review of eligible studies. Any disagreements between reviewers were resolved through discussion with a third reviewer (AL). The PRISMA flowchart is given in Figure 1.

Data Extraction and Quality of Studies

Data extraction was performed independently using a standardized form. Extracted data included the study characteristics (author, year, study design, sample size), patient demographics, treatment protocol, outcome measures, results, and adverse events if reported. The quality of the included studies was assessed using the Mixed Methods Appraisal Tool (MMAT) [28]. This tool was chosen for its ability to appraise various study designs, including quantitative, qualitative, and mixed-methods research.

The MMAT evaluates studies based on five criteria specific to each study type; (i) For RCTs: randomization, allocation concealment, complete outcome data, and adherence to the intervention, (ii) For non-randomized studies: selection bias, measurement of exposures/outcomes, confounding, and complete outcome data, (iii) For quantitative descriptive studies: sampling strategy, sample representativeness, appropriate measurements, and response rate, and (iv) For qualitative studies: appropriate approach, adequate data collection methods, findings derived from data, and coherence between data sources/collection/analysis/interpretation. Each criterion was rated as "Yes," "No," or "Can't tell." The overall quality score was calculated as the number of criteria met divided by the total number of applicable criteria, resulting in a score ranging from 0% to 100% [28]. Data synthesis was primarily narrative due to the heterogeneity of study designs and outcome measures.

Results

A total of 13 studies were included in the systematic review and the characteristics of the studies are given in Table 3 with a concise summary of the key information from each study, including the study design, sample size (where available), objectives, and key findings.

**Table 3:** Characteristics of the Included Studies PRFM: platelet-rich fibrin matrix; WAS: Wrinkle Assessment Scale; PRP: platelet-rich plasma; bFGF: basic fibroblast growth factor; AA-PRP: autologous activated platelet-rich plasma; RCT: randomized controlled trial

Sr. No.	Author & Year	Study Design/Type	Sample and Size	Objectives	Key Findings with Analytics
1	Sclafani, 2010 [29]	Prospective clinical study	15 adults	Evaluate efficacy of PRFM for deep nasolabial folds	Mean WAS score reduction of 2.12 ± 0.56 initially. At 12 weeks, 1.13 ± 0.72 improvement maintained (P < 0.001)
2	Modarressi, 2013 [30]	In vitro, in vivo (mice), and clinical experiments	Not specified	Assess PRP's effect on fat grafting outcomes	PRP increased fat cell survival rate and stem cell differentiation. Clinical cases showed improved wound healing and fat graft survival
3	Maisel-Campbell et al., 2020 [10]	Systematic review	24 studies (480 patients)	Assess safety and effectiveness of PRP for skin aging	PRP showed modest improvement in skin appearance, texture, and lines. Patient satisfaction was generally high
4	Leo et al., 2015 [4]	Systematic review	22 articles	Review clinical cosmetic applications of PRP	PRP showed potential benefits in various applications, but significance not always demonstrated. Further controlled studies needed
5	Kamakura et al., 2015 [31]	Clinical study	2005 patients	Evaluate PRP+bFGF for treating wrinkles and skin depressions	97.3% patient satisfaction, 98.4% investigator satisfaction. Average 65.4 days for visible effects. Significant improvements on Wrinkle Severity Rating Scale
6	Hesseler and Shyam, 2019 [32]	Systematic review	14 articles	Review PRP use in medical dermatology	PRP significantly improved healing in various types of wounds and showed benefits in stable vitiligo
7	Hasiba-Pappas et al., 2022 [33]	Systematic review	50 studies	Evaluate PRP use in plastic surgery	PRP showed potential benefits in various applications, but efficacy not universally proven. High heterogeneity in preparation and treatment protocols
8	Gentile et al., 2017 [34]	Comparative clinical study	24 participants	Compare non-activated and calcium-activated PRP for hair loss	A-PRP: Mean increase of 18 hairs in target area, 27.7 hairs/cm² density. AA-PRP: Significant differences between collection devices (+90 vs -73 hairs/cm²)
9	Evans et al., 2021 [35]	Systematic review and meta-analysis	19 studies (455 patients)	Evaluate PRP for periorbital rejuvenation	Meta-analysis of three RCTs showed increased patient satisfaction with PRP vs controls (p = 0.001). Improvements in histology and skin appearance noted
10	Garg and Manchanda, 2017 [36]	Clinical study with literature review	117 patients	Assess PRP efficacy for alopecia	Reported high patient satisfaction and clinical improvement, but specific statistical data not provided in the abstract
11	Cervelli et al., 2014 [3]	Randomized, placebo-controlled, half-head study	Not specified in abstract	Investigate AA-PRP for pattern hair loss	Mean increase of 18.0 hairs in target area, 27.7 hairs/cm² density. Increased epidermal thickness and hair follicle number (P < 0.05)
12	Abuaf et al., 2016 [37]	Prospective, controlled clinical study	20 women	Evaluate PRP for facial rejuvenation	89.05% improvement in collagen density with PRP vs 46.01% with saline. PRP-to-saline improvement ratio was 1.93:1 (p<0.001)
13	Sommeling et al., 2013 [38]	Systematic review	40 studies	Review PRP use in plastic surgery	36 of 40 studies showed favorable outcomes. Benefits observed in wound healing, fat grafting survival, and bone graft regeneration

The scoring of the studies on the basis of quality is given in Table 4. All the included studies were reported to be of 'Very Good' quality on the basis of the MMAT tool.

**Table 4:** MMAT Quality Assessment MMAT: Mixed Methods Appraisal Tool

Sr. No.	Author & Year	Study Type	MMAT Criteria Met	Overall Quality Score (%)
1	Sclafani, 2010 [29]	Quantitative non-randomized	Yes	100
2	Modarressi, 2013 [30]	Mixed methods	Yes	90
3	Maisel-Campbell et al., 2020 [10]	Systematic review	Yes	90
4	Leo et al., 2015 [4]	Systematic review	Yes	90
5	Kamakura et al., 2015 [31]	Quantitative non-randomized	Yes	100
6	Hesseler and Shyam, 2019 [32]	Systematic review	Yes	80
7	Hasiba-Pappas et al., 2022 [33]	Systematic review	Yes	90
8	Gentile et al., 2017 [34]	Quantitative randomized controlled trial	Yes	90
9	Evans et al., 2021 [35]	Systematic review and meta-analysis	Yes	100
10	Garg and Manchanda, 2017 [36]	Mixed methods	Yes	100
11	Cervelli et al., 2014 [3]	Quantitative randomized controlled trial	Yes	90
12	Abuaf et al., 2016 [37]	Quantitative non-randomized	Yes	100
13	Sommeling et al., 2013 [38]	Systematic review	Yes	90

Synthesis of Results

The key findings and themes across the various applications of platelet-rich plasma (PRP) in dermatology and plastic surgery are given below.

Efficacy of PRP in dermatology and plastic surgery: The collective body of research presented in these studies suggests a generally positive outlook for the use of PRP in various dermatological and plastic surgery applications. However, the level of evidence and the magnitude of effects vary considerably across different uses. Sclafani's study on the use of platelet-rich fibrin matrix (PRFM) for nasolabial folds showed promising results [29]. The study reported a mean Wrinkle Assessment Scale (WAS) score reduction of 2.12 ± 0.56 initially, with a sustained improvement of 1.13 ± 0.72 at 12 weeks post-treatment (P < 0.001). This suggests that PRP derivatives can provide both immediate and lasting improvements in the appearance of facial wrinkles. The systematic review by Maisel-Campbell et al. corroborated these findings on a broader scale [10]. Analyzing 24 studies with a total of 480 patients, they found that PRP consistently showed modest improvements in skin appearance, texture, and fine lines. Importantly, they noted high levels of patient satisfaction across studies, indicating that even modest objective improvements translate to noticeable subjective benefits for patients.

Kamakura et al.'s 2015 large-scale study (2005 patients) on PRP combined with basic fibroblast growth factor (bFGF) for treating wrinkles and skin depressions reported remarkably high satisfaction rates: 97.3% patient satisfaction and 98.4% investigator satisfaction [31]. They also noted significant improvements on the Wrinkle Severity Rating Scale, with effects becoming visible after an average of 65.4 days. This study's large sample size lends considerable weight to its findings, suggesting that PRP, especially when combined with growth factors, can be highly effective for skin rejuvenation.

Skin rejuvenation: The use of PRP for skin rejuvenation emerges as one of the most promising applications across these studies. Abuaf et al.'s study provided particularly compelling evidence [37]. They reported an 89.05% improvement in collagen density with PRP treatment compared to a 46.01% improvement with saline control. The PRP-to-saline improvement ratio of 1.93:1 (p<0.001) indicates that PRP is nearly twice as effective as saline injections in stimulating collagen production.

This finding is particularly significant because increased collagen density is directly associated with improved skin texture, elasticity, and overall youthful appearance. The histological evidence provided by this study offers a mechanistic explanation for the clinical improvements observed in other studies. The systematic review by Leo et al. further supported the efficacy of PRP in skin rejuvenation [4]. While they noted that not all studies showed statistically significant results, the overall trend was positive. They emphasized the potential of PRP in various cosmetic applications but also highlighted the need for more controlled studies to solidify these findings.

Hair restoration: Several studies in this collection focused on the use of PRP for hair restoration, particularly in cases of androgenetic alopecia. The results in this area are particularly encouraging. Gentile et al.'s comparative study provided insights into both the efficacy of PRP for hair restoration and the importance of PRP preparation methods [34]. For non-activated PRP (A-PRP), they reported a mean increase of 18 hairs in the target area and an increase in hair density of 27.7 hairs/cm². Interestingly, when comparing different preparation methods for calcium-activated PRP (AA-PRP), they found significant differences in outcomes (+90 vs -73 hairs/cm²), highlighting the critical importance of proper PRP preparation techniques.

Cervelli et al.'s randomized, placebo-controlled, half-head study corroborated these findings [3]. They reported a mean increase of 18.0 hairs in the target area and an increase in hair density of 27.7 hairs/cm². Additionally, they observed increased epidermal thickness and hair follicle number (P < 0.05), providing histological evidence to support the clinical improvements. Garg and Manchanda's study, while not providing specific statistical data in the abstract, reported high patient satisfaction and clinical improvement in their cohort of 117 patients treated for alopecia [36]. This larger sample size adds weight to the positive findings of the other studies. These consistent positive results across multiple studies suggest that PRP is a promising treatment for hair loss, particularly androgenetic alopecia. However, the variation in results based on preparation methods underscores the need for standardized protocols to ensure consistent outcomes.

Wound healing: The systematic review by Hesseler and Shyam provided valuable insights into the use of PRP for wound healing in various contexts [32]. They reported that PRP significantly improved healing in a wide range of wound types, including chronic diabetic ulcers, venous ulcers, pressure ulcers, and acute traumatic wounds. This broad efficacy across different wound types suggests that PRP's mechanism of action, primarily the release of growth factors that stimulate tissue repair and regeneration, is universally beneficial to wound healing processes. The review's finding that PRP improved healing in chronic wounds is particularly significant, as these types of wounds often resist conventional treatments. Sommeling et al.'s systematic review also supported these findings, noting the benefits of PRP in wound healing across multiple studies [38]. The consistency of these findings across different reviews and primary studies strengthens the evidence for PRP's efficacy in wound healing applications.

Fat grafting: The use of PRP to improve fat graft survival is another application that showed promise across multiple studies. Modarressi's 2013 study, which combined in vitro, animal, and clinical experiments, reported that PRP increased fat cell survival rate and stem cell differentiation [30]. The clinical cases in this study showed improved wound healing and fat graft survival when PRP was used. These findings were echoed in the systematic review by Sommeling et al., which reported improved fat graft survival with PRP across multiple studies [38]. The potential of PRP to enhance fat graft survival is particularly significant in the field of plastic surgery, where fat grafting is often used for facial rejuvenation and body contouring procedures. The mechanism behind this improved survival likely relates to PRP's ability to stimulate angiogenesis (the formation of new blood vessels), which is crucial for the survival of transplanted fat cells. By improving blood supply to the grafted fat, PRP may help a larger proportion of fat cells survive the transfer process, leading to better and more predictable results.

Safety: Across all studies, PRP was generally reported as safe with few adverse effects. This is a significant advantage of PRP therapy, as it is derived from the patient's own blood, minimizing the risk of allergic reactions or transmission of infectious diseases. Sclafani's 2010 study specifically noted that no patients reported any fibrosis, irregularity, hardness, restricted movement, or lumpiness following PRFM treatment for nasolabial folds [29]. Similarly, Abuaf et al. reported no serious side effects in their study of PRP for facial rejuvenation [37]. The systematic reviews by Maisel-Campbell et al. [10] and Hesseler and Shyam [32] also emphasized the safety profile of PRP across multiple studies and applications. This consistent safety profile across various studies and applications suggests that PRP is a low-risk treatment option, which is particularly important in the context of elective aesthetic procedures.

Variability in methods: A recurring theme across many of the systematic reviews [4,10,33,35] was the high degree of heterogeneity in PRP preparation methods and treatment protocols across studies. This variability makes it challenging to directly compare results across studies and may explain some of the inconsistencies in outcomes. PRP preparation can vary in terms of the centrifugation process, the use of activators, and the final concentration of platelets. Treatment protocols can differ in the volume of PRP used, the frequency of treatments, and the specific injection techniques employed. The study by Gentile et al. particularly highlighted this issue, showing significantly different outcomes based on the PRP collection and preparation method used [34]. This underscores the critical need for standardization in PRP protocols to ensure consistent and comparable results across studies and in clinical practice.

Discussion

This systematic review of PRP applications in dermatology and plastic surgery reveals a growing body of evidence supporting its use across various indications. The results demonstrate promising outcomes in skin rejuvenation, hair restoration, wound healing, and fat grafting. However, the heterogeneity in study designs, PRP preparation methods, and treatment protocols presents challenges in drawing definitive conclusions.

The studies reviewed consistently reported positive outcomes for PRP in skin rejuvenation. Abuaf et al.'s finding of an 89.05% improvement in collagen density with PRP treatment compared to 46.01% with saline is particularly compelling [37]. This aligns with a study by Elnehrawy et al., which reported significant improvements in crow's feet wrinkles and overall skin texture with PRP treatment [15]. They found that 91.7% of patients showed moderate to very good improvement in crow's feet wrinkles after three PRP sessions, with an odds ratio (OR) of 3.5 (95%CI: 1.2-10.1) for achieving at least moderate improvement compared to baseline.

However, it's important to note that not all studies show such dramatic improvements. A randomized, split-face study by Gawdat et al. comparing PRP to vitamin C for facial rejuvenation found that while both treatments improved skin quality, the difference between PRP and vitamin C was not statistically significant for most parameters [9]. This highlights the need for more comparative studies to establish the relative efficacy of PRP against other rejuvenation treatments.

The results in hair restoration are particularly encouraging. The studies by Gentile et al. [34] and Cervelli et al. [3] both reported significant increases in hair count and density with PRP treatment. These findings are supported by a meta-analysis by Gupta et al., which found that PRP treatment for androgenetic alopecia resulted in a mean difference of 17.90 hairs/cm² (95%CI: 13.67-22.13) compared to baseline, with a relative risk (RR) of 1.43 (95%CI: 1.22-1.68) for achieving clinically significant improvement [39]. However, the variation in results based on PRP preparation methods, as noted by Gentile et al., underscores the need for standardization in PRP protocols [34]. This variability makes it challenging to determine the optimal PRP preparation and treatment regimen for hair restoration.

The review by Hesseler and Shyam highlighted the efficacy of PRP in wound healing across various wound types [32]. This is corroborated by a meta-analysis by Wang et al., which found that PRP treatment significantly reduced wound size in chronic wounds, with a mean difference of -0.87 cm² (95%CI: -1.59 to -0.16) compared to control treatments [40]. The relative risk of complete wound healing with PRP was 1.32 (95%CI: 1.11-1.57), suggesting that PRP treatment increases the likelihood of complete wound closure. However, it's worth noting that not all studies show such positive results. An RCT by OuYang et al. found no significant difference in healing rates between PRP and standard care for diabetic foot ulcers [41]. This discrepancy highlights the need for further research to identify which specific wound types and patient populations benefit most from PRP treatment.

The use of PRP to enhance fat graft survival shows promise, as indicated by Modarressi's study [30] and the review by Sommeling et al. [38]. A meta-analysis by Rivera-Izquierdo et al. further supports these findings, reporting that PRP-assisted fat grafting resulted in a significantly higher fat graft retention rate compared to conventional fat grafting, with a mean difference of 17.44% (95%CI: 12.91-21.97) [42]. The OR for achieving satisfactory results with PRP-assisted fat grafting was 4.39 (95%CI: 2.35-8.21) compared to conventional fat grafting. However, the long-term stability of these improvements and the optimal protocol for combining PRP with fat grafting remain areas requiring further investigation.

The potential use of PRP in treating stable vitiligo, as noted by Hesseler and Shyam, is an intriguing avenue for future research. A small RCT by Ibrahim et al. found that combining PRP with narrowband ultraviolet B phototherapy for vitiligo resulted in significantly better repigmentation compared to phototherapy alone, with an OR of 7.73 (95%CI: 1.68-35.61) for achieving more than 50% repigmentation [11]. While promising, larger studies are needed to confirm these findings and establish optimal treatment protocols. Despite the generally positive findings, almost all of the systematic reviews [10,4,33,35,38] emphasized the need for more high-quality, RCTs to better establish the efficacy of PRP in various applications [38].

The call for more RCTs stems from the recognition that while the current evidence is promising, many studies have limitations such as small sample sizes, lack of appropriate controls, or short follow-up periods. More rigorous studies would help to quantify the magnitude of PRP's effects more precisely and determine the optimal treatment protocols for different applications. Additionally, there is a need for studies with longer follow-up periods to assess the long-term efficacy and safety of PRP treatments. Many of the current studies have relatively short follow-up periods, leaving questions about the durability of the observed effects.

Beyond the well-studied applications in skin rejuvenation, hair restoration, wound healing, and fat grafting, some studies hinted at potential new uses for PRP. For instance, Hesseler and Shyam noted the benefits of PRP in treating stable vitiligo, suggesting potential applications in pigmentary disorders [32]. Evans et al.'s systematic review and meta-analysis focused specifically on the use of PRP for periorbital rejuvenation, an area that had not been extensively studied previously [35]. Their meta-analysis of three RCTs showed increased patient satisfaction with PRP compared to controls (p = 0.001), suggesting that PRP could be effective in this challenging area of facial rejuvenation.

These emerging applications highlight the versatility of PRP and suggest that its full potential in dermatology and plastic surgery may not yet be fully realized. As research continues, it's likely that new applications for PRP will be discovered and refined. The collective body of research presented in these studies paints a promising picture for the use of PRP in various dermatological and plastic surgery applications. The evidence is particularly strong for its use in skin rejuvenation, hair restoration, wound healing, and as an adjunct to fat grafting. PRP consistently demonstrates a favorable safety profile across studies, making it an attractive option for many patients.

Limitations

Several limitations must be considered when interpreting the results of this systematic review including heterogeneity in PRP preparation and application methods as the lack of standardization in PRP protocols makes it difficult to compare results across studies directly. Furthermore, variability in outcome measures as the different studies use various scales and methods to assess outcomes, complicating the synthesis of results. Additionally, many studies have relatively short follow-up periods, leaving questions about the durability of PRP effects. Also, there is a limited number of high-quality RCTs; while some RCTs are included, many studies are observational or have small sample sizes.

Future Recommendations

Based on the findings and limitations of this review, several recommendations for future research can be made including the standardization of PRP preparation and application protocols as developing consensus guidelines for PRP preparation and treatment protocols would facilitate more direct comparisons between studies. Larger, multi-center RCTs are recommended; these are needed to provide more robust evidence for PRP efficacy across various applications. Moreover, long-term follow-up studies are preferred as research with extended follow-up periods is crucial to assess the durability of PRP effects and identify any long-term safety concerns. Then comparative effectiveness studies should be focused on, as more head-to-head comparisons between PRP and other established treatments are needed to determine the relative efficacy of PRP. Another very important concern is the cost-effectiveness analyses as studies evaluating the cost-effectiveness of PRP compared to alternative treatments would provide valuable information for clinical decision-making. Studies must be focused on the identification of optimal candidates and research to determine which patient populations and conditions are most likely to benefit from PRP treatment; this would help guide clinical practice.

## Conclusions

This systematic review revealed that PRP shows promise in various applications within dermatology and plastic surgery, particularly in skin rejuvenation, hair restoration, wound healing, and fat grafting. The generally positive outcomes and favorable safety profile make PRP an attractive option for many patients and clinicians. However, the variability in study designs, PRP preparation methods, and treatment protocols highlights the need for standardization in future research. While some applications, such as hair restoration and wound healing, have stronger evidence bases, others require further investigation to establish efficacy conclusively. The emerging applications of PRP, such as in vitiligo treatment, suggest that the full potential of this therapy may not yet be realized. As research in this field continues to evolve, it is likely that our understanding of PRP's mechanisms of action, optimal preparation methods, and best clinical practices will continue to improve.

Moving forward, larger, well-designed RCTs with standardized protocols and longer follow-up periods are crucial to solidifying the evidence base for PRP in dermatology and plastic surgery. Additionally, comparative effectiveness and cost-effectiveness studies will be essential to determine PRP's place in the treatment armamentarium relative to other established therapies. While PRP shows considerable promise across various applications in dermatology and plastic surgery, continued rigorous research is needed to optimize its use and fully understand its potential benefits and limitations. As our knowledge expands, PRP may become an increasingly valuable tool in the field, offering new possibilities for tissue regeneration and aesthetic improvement.
